# ROLE OF PLACE AS A DETERMINANT OF PARENT–ADULT CHILD GEOGRAPHIC PROXIMITY AND CAREGIVING

**DOI:** 10.1093/geroni/igad104.0188

**Published:** 2023-12-21

**Authors:** Stipica Mudrazija, Elizabeth Peters, Fernando Hernandez Lepe

**Affiliations:** University of Washington, Seattle, Washington, United States; Urban Institute, Washington, District of Columbia, United States; Urban Institute, Washington, District of Columbia, United States

## Abstract

While research has shown that the need for care impacts how close parents and adult children live, the relative importance of various factors shaping parent-child geographic proximity remains unclear, especially as it relates to characteristics of their geographic locations and communities. We explore variation in and determinants of the geographic proximity of older parents and adult children, conditional on parents needing care. Using pooled 2004-2014 data from the Health and Retirement Study on respondents age 65 years or older and their children, alongside detailed county-level contextual information from various sources, we analyze the importance of various determinants of parent-child geographic proximity, including parental health, age, and other personal characteristics, children’s personal characteristics, parent-child dyad characteristics, and place-specific characteristics that relate with availability of different health and aging services, housing affordability, and labor market opportunities. Preliminary regression results show that coresidence is substantially less likely in areas with higher availability of nursing care and continuing care options. Among non-coresidents, living in close proximity (distances under 10 miles) is less likely if parent’s area has more nursing care options, but more likely if health services that support living in place are available. Additionally, local economic and housing conditions play a role, with higher median household income associated with children living closer to parents and higher cost of rent with living further. Place-specific characteristics are important determinants of parent-child coresidence and proximity, and policymakers and care providers should consider them when determining the most effective ways to support older adults who need care.

